# Editorial: New avenues for the development of advanced immunotherapies: capitalizing on studies of the B and T cell receptor repertoire

**DOI:** 10.3389/fimmu.2025.1693587

**Published:** 2025-10-02

**Authors:** Fabio Nicolini, Mar Naranjo Gomez, Mireia Pelegrin, Anna De Lucia, Lucia Mazzotti, Massimiliano Mazza

**Affiliations:** ^1^ Advanced Cellular Therapies and Rare Tumors Unit, Istituto Di Ricovero E Cura A Carattere Scientifico Istituto Romagnolo per lo Studio dei Tumori (IRST) “Dino Amadori”, Meldola, Italy; ^2^ IRMB, Univ Montpellier, Institut National de la Santé et de la Recherche Médicale, CHU Montpellier, CNRS, Montpellier, France

**Keywords:** immunotherapy, bi-specific antibodies, CAR-T cells, TCR, BCR repertoire

Immunotherapy has transformed the landscape of cancer treatment and other medical fields by utilising the power of the immune system through immune checkpoint inhibitors, adoptive cell therapies, and monoclonal antibodies. Despite significant progress, many promising therapeutic and diagnostic targets remain underexplored in clinical practice. Recent innovations, based on studies of the B and T cell receptor repertoires—particularly using NSG-based technologies—have opened the way for next-generation immunotherapies—including CAR therapies, engineered antibodies, and bispecific antibodies—broadening treatment options for cancer, autoimmune diseases, and infectious disorders. This Research Topic introduces the collection of articles published in Frontiers in Immunology: Cancer Immunity and Immunotherapy Research Topic, dedicated to exploring these advanced approaches, with a focus on the translational potential of immune receptor repertoire analysis and antibody development to shape the future of precision immunotherapy.

In particular, the review by Haus-Cohen and Reiter emphasises that conventional immunotherapies targeting surface antigens only address a small part of the cancer proteome, thereby limiting their therapeutic potential. Notably, the majority of intracellular proteins are processed and presented on tumour cell surfaces as peptide-MHC class I complexes, representing a vast and largely untapped resource for immunotherapeutic intervention. The review comprehensively outlines (1) innovative approaches leveraging T-cell receptors (TCRs) and TCR-like (TCRL) antibodies to recognise and target these intracellularly derived peptide-MHC complexes. Emerging therapeutic modalities—including TCR-engineered T cells, CAR-T cells directed against peptide-MHC complexes, and bispecific T cell engagers—have shown promising clinical efficacy, as exemplified by recent FDA approvals for treatments of uveal melanoma and synovial sarcoma. These developments mark a paradigm shift towards exploiting the intracellular proteome to expand the arsenal of cancer immunotherapies.

The study by Snyder et al. reports a large-scale, high-resolution analysis of SARS-CoV-2-specific T-cell responses across over 1,400 individuals, identifying more than 70,000 TCRs linked to viral antigens through MIRA (1, 2) and repertoire sequencing (3). The study characterizes the breadth, magnitude, immunodominance, and persistence of T-cell immunity across diverse demographics and disease stages. Their findings demonstrate robust and durable adaptive immunity, establishing TCR-based molecular diagnostics with superior early sensitivity. This work highlights the critical role of TCR repertoire profiling in understanding immune protection and informs the development of innovative immunotherapies targeting viral infections.

The study by Nolan et al. presents the ImmuneCODE database, a comprehensive resource comprising 1,486 deeply sequenced T-cell receptor beta (TCRβ) repertoires and more than 160,000 high-confidence SARS-CoV-2-associated TCRs. By integrating data from natural and synthetic exposures, the database maps TCR specificity to viral epitopes, providing unprecedented insights into adaptive immunity. Freely accessible, ImmuneCODE supports research, diagnostics, vaccine development, and TCR-antigen prediction tools (1, 4). This resource exemplifies the power of large-scale, high-resolution TCR repertoire profiling to advance immunotherapy and deepen understanding of viral immune responses, positioning it as a foundation for future T-cell-mediated immunity research and therapeutic design.

The article from Zhang et al. examines why some patients with advanced oesophageal squamous cell carcinoma respond better than others to a regimen combining camrelizumab, an anti–PD-1 antibody, with platinum-based chemotherapy. Using immune repertoire sequencing, the researchers found that responders displayed broader and more diverse T-cell receptor profiles, suggesting a stronger ability to recognise tumour antigens. Importantly, longitudinal analyses revealed dynamic changes in these repertoires during treatment, further distinguishing responders from non-responders. These insights highlight how immune signatures can predict outcomes (5) and underscore the promise of repertoire sequencing as a tool for tailoring immunotherapy in precision oncology (6).

Recent work by Nicolini et al. investigating the B-cell receptor (BCR) repertoires of metastatic melanoma (MM) patients, particularly those exhibiting favourable responses to first-line anti-PD-1 therapy (7), provides valuable insights into the humoral mechanisms driving effective immunotherapy. Utilizing advanced sequencing technologies (8), the researchers isolated circulating memory B cells from well-defined groups of non-responders, partial responders, and complete responders to nivolumab. Through comprehensive BCR repertoire analysis, they tracked the emergence and evolution of complementarity-determining region 3 sequences pre- and post-immune checkpoint inhibitor (ICI) treatment, with an emphasis on *de novo* clonotypes. This targeted exploration of the adaptive immune response facilitated the recombinant production of fully human antibodies assessed for tumour specificity using melanoma cell lines, patient-derived xenograft models, and tissue microarrays. These findings illuminate a path toward personalised, antibody-based interventions in melanoma, driven by the real-time evolution of the patient’s own immune system.

Zhang et al. uncover an unexpected connection between ovarian cancer and the Epstein-Barr virus (EBV). Through the examination of tumour and ascites samples, the research team identified rare B cells that not only generated antibodies but also harboured EBV in a latent form. Interestingly, these cells spontaneously became immortalised *in vitro* while retaining their ability to secrete antibodies. The antibodies targeted proteins related to tumours, including CCDC155, PDP2, and GRB2, with anti-CCDC155 antibodies found in nearly half of the blood samples from patients. These discoveries provide insight into how EBV-infected B cells influence the tumour microenvironment (9) and could potentially lead to new immunotherapy approaches for ovarian cancer (10).

The article by Musnier et al., “AI-enhanced profiling of phage-display-identified anti-TIM3 and anti-TIGIT novel antibodies” presents a powerful integration of computational and experimental immunology. The authors introduce a pipeline that combines early-stage phage display with AI-driven analysis to assess the developability and epitope specificity of antibody candidates against the TIM3 and TIGIT immune checkpoints (11). From 5 leads with similar binding, two were flagged for poor developability, while the remaining three underwent structural modelling, revealing predicted antagonistic interactions with targets, demonstrating how AI can enhance early decision-making in antibody discovery, thereby reducing late-stage failures and accelerating therapeutic development.

This multi-centre, prospective cohort study by X. Wang and colleagues evaluated the efficacy and safety of camrelizumab combined with platinum-based chemotherapy (TP or FP) as a first-line treatment for advanced oesophageal squamous cell carcinoma (ESCC) and explored treatment response mechanisms using immune repertoire sequencing (IRS). IRS reflects the diversity of T and B lymphocytes at a given time, indicating the immune system’s ability to respond to external stimuli (12, 13). A total of 88 patients received camrelizumab plus TP or FP, demonstrating that this regimen is effective and well-tolerated. Additionally, IRS revealed distinct T-cell receptor (TCR) and B-cell receptor (BCR) features between responders and non-responders. Responders showed greater immune diversity with enrichment of specific T-cell clones, suggesting a more targeted antitumor response. Similarly, differences in IGH-CDR3 amino acid composition and clonotype abundance suggest that certain B-cell clones may play a key role in tumour recognition. These immune repertoire characteristics hold potential as predictive biomarkers and warrant further validation in larger cohorts (14).

The original article published by Mohan et al. is a comparative study of T-cell-engaging bispecific antibodies (BsAbs) targeting EGFR and CD3, examining the IgG-based DVD-Ig and non-IgG-based BiTE formats, highlighting crucial distinctions for cancer immunotherapy. Both BsAb formats demonstrated effective dual antigen binding and robust induction of T-cell-mediated cytotoxicity *in vitro*. Notably, BiTEs exhibited stronger and faster tumour cell killing than DVD-Igs, although both formats achieved significant cytotoxicity after extended incubation periods. The Fc region present in DVD-Igs was linked to delayed and reduced T-cell killing potency compared to the Fc-less BiTEs, impacting cytokine and granzyme B release. However, DVD-Igs offered greater manufacturing yields and stability, important for clinical translation given the complex nature and production challenges of BsAbs (15). The study also emphasizes that better CD3 binding does not always enhance cytotoxic function, underscoring the need to carefully balance affinity and stability in antibody engineering. Such insights are essential as the field advances toward safer, more effective bispecific therapeutics for solid tumours (16).

Collectively, these studies illustrate the dynamic progress in immunotherapy, propelled by enhanced molecular and cellular comprehension of T-cell and B-cell receptor repertoires, intracellular antigen targeting, and antibody engineering. They highlight how leveraging the specificity and diversity of adaptive immunity not only improves therapeutic effectiveness against cancer and viral pathogens but also contributes to the development of innovative diagnostic and predictive instruments. As the integration of immune repertoire profiling and novel immunotherapeutic strategies continues to advance, personalised medicine approaches are set to become more precise, effective, and enduring, establishing new benchmarks for future clinical interventions.
